# Mutation of *ACX1*, a Jasmonic Acid Biosynthetic Enzyme, Leads to Petal Degeneration in Chinese Cabbage (*Brassica campestris* ssp. *pekinensis*)

**DOI:** 10.3390/ijms20092310

**Published:** 2019-05-10

**Authors:** Shenling Peng, Shengnan Huang, Zhiyong Liu, Hui Feng

**Affiliations:** Department of Horticulture, Shenyang Agricultural University, 120 Dongling Road, Shenhe District, Shenyang 110866, China; Shenlinggogo@163.com (S.P.); hsn870806@163.com (S.H.); lzyky99@163.com (Z.L.)

**Keywords:** Chinese cabbage, petal, mutation, jasmonic acid, acyl-CoA oxidase1

## Abstract

Petal color, size, and morphology play important roles in protecting other floral organs, attracting pollinators, and facilitating sexual reproduction in plants. In a previous study, we obtained a petal degeneration mutant (*pdm*) from the ‘FT’ doubled haploid line of Chinese cabbage and found that the candidate gene for *pdm*, *Bra040093*, encodes the enzyme acyl-CoA oxidase1. In this study, we sought to examine the gene networks regulating petal development in *pdm* plants. We show that the mRNA and protein expression of *Bra040093*, which is involved in the jasmonic acid (JA) biosynthetic pathway, were significantly lower in the petals of *pdm* plants than in those of ‘FT’ plants. Similarly, the JA and methyl jasmonate (MeJA) contents of petals were significantly lower in *pdm* plants than in ‘FT’ plants and we found that exogenous application of these hormones to the inflorescences of *pdm* plants restored the ‘FT’ phenotype. Comparative analyses of the transcriptomes of ‘FT’, *pdm* and *pdm* + JA (pJA) plants revealed 10,160 differentially expressed genes (DEGs) with consistent expression tendencies in ‘FT’ vs. *pdm* and pJA vs. *pdm* comparisons. Among these DEGs, we identified 69 DEGs related to floral organ development, 11 of which are involved in petal development regulated by JA. On the basis of qRT-PCR verification, we propose regulatory pathways whereby JA may mediate petal development in the *pdm* mutant. We demonstrate that mutation of *Bra040093* in *pdm* plants leads to reduced JA levels and that this in turn promotes changes in the expression of genes that are expressed in response to JA, ultimately resulting in petal degeneration. These findings thus indicate that JA is associated with petal development in Chinese cabbage. These results enhance our knowledge on the molecular mechanisms underlying petal development and lay the foundations for further elucidation of the mechanisms associated with floral organ development in Chinese cabbage.

## 1. Introduction

In plants, floral organs play essential roles in sexual reproduction [1,2]. The morphological development of floral organs is a highly complex and inherently stable process that is an indispensable facet of plant growth and development. The process is regulated by the expression of multiple genes and Coen and Meyerowitz [3] have identified many functional genes related to the regulation of floral organ development in homeotic mutants of model plants, including *Arabidopsis thaliana* and *Antirrhinum majus*, on the basis of which they proposed the ABC model of floral organ development. Subsequently, ABCD [4], ABCDE [5], and tetramer [6] models were proposed to explain these regulatory processes and these studies have accordingly stimulated further research on the molecular mechanisms underlying the evolution of floral organs.

Although petals are not directly involved in reproduction, they play critical roles in attracting pollinators and protecting the reproductive organs to ensure pollination and fertilization at the flowering stage [7]. Petals are platy structures, the size of which is regulated by a number of transcription factors and plant hormones. In *Arabidopsis thaliana*, *AINTEGUMENTA* (*ANT*) encodes an AP2-domain family transcription factor that acts downstream of *AUXIN-REGULATED GENE INVOLVED IN ORGAN SIZE* (*ARGOS*), and overexpression of *ANT* results in increases in the size of leaves, inflorescences, and floral organs [8]. *KLUH* (*KLU*) encodes cytochrome P450 monooxygenase CYP78A5 [9,10] and the loss-of-function mutant *klu* shows early termination of mitosis, which leads to smaller-sized petals and sepals [11,12], compared with those observed in normal plants. *BIGPETALp* (*BPEp*), a basic helix-loop-helix (*bHLH*) transcription factor, is preferentially expressed in petals. Expression of the *BPEp* gene is regulated by jasmonic acid (JA), which affects cell elongation in petals [13,14]. Studies on the double mutant *bpe-arf8* have shown that, at the early stage of petal development, *BPEp* and *Auxin response factors8* (*ARF8*) synergistically limit mitotic cell growth, whereas at the late stage of petal development, the proteins encoded by these genes interact to limit cell expansion, thereby regulating petal size [15,16].

JA and its derivatives, which include methyl jasmonate (MeJA) and amino acid conjugates, are together called jasmonates (JAs). The CORONATINE INSENSITIVE1 (COI1) is the receptor of JA [17,18]. The hormone JA has extensive physiological effects in plants, including the induction of defense-related gene expression [19,20,21,22] and the regulation of plant growth and development, including root growth [23,24], floral organ development [25,26,27,28], and leaf senescence [29,30]. In rice (*Oryza sativa*), the JA biosynthesis mutant *extra glumel3* (*egl3*) is characterized by changes in the number and properties of floral organs and the floral meristem is unable to terminate growth normally. However, exogenously applied JA can partially recover floral development in *egl3* [31,32]. In oilseed rape (*Brassica napus*), exogenous application of MeJA accelerates the flowering time and affects floral organ development [33], whereas in *Arabidopsis thaliana*, JA acts as a morphogenesis signal that regulates the processes of cell expansion and petal growth [25] and mainly participates in the regulation of late-stage floral organ development [13]. The gene defective in anther dehiscence1 (*DAD1*) encodes particular phospholipase A1 (*PLA1*), which represents the primary step of JA biosynthesis [27], inducing the upper region of filaments to produce JA, which in turn regulates petal opening and anther dehiscence at the appropriate time. The *R2R3-MYB* transcription factors *MYB21* and *MYB24* function as key regulators in the process of stamen development induced by JA, and *myb21* and *myb24* double mutants have been observed to produce short filaments, anthers that fail to dehisce, and petals that are unable to open [34,35,36].

β-oxidation plays an important role in the biosynthesis of JA [37]. Acyl-CoA oxidase (ACX) is the first and the key step controlling enzyme involved in fatty acid β-oxidation. *ACX1* orthologs have been extensively studied in many plant species, mainly including tomato (*Lycopersicon esculentum*), *Arabidopsis*, soybean (*Glycine max*), and tea (*Camellia sinensis*) [38,39,40,41].

**Figure 1:**
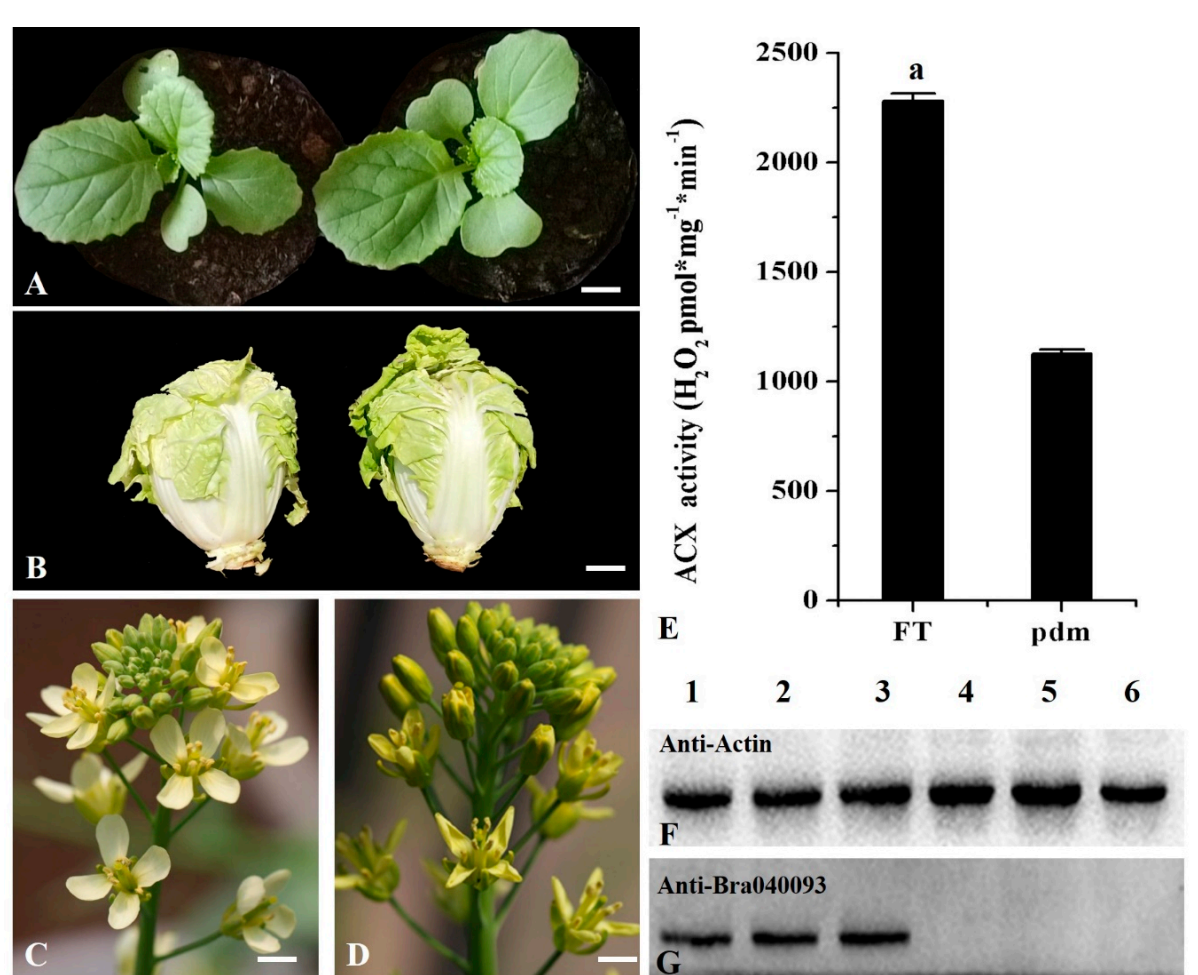
Phenotypes of ‘FT’ and *pdm* plants and analysis of *Bra040093* expression patterns. (**A**–**D**) Phenotypes of ‘FT’ and *pdm* plants. ((**A**) at the seedling stage, (**B)** at the heading stage, and (**C**,**D**) at the flowering stage. Left: ‘FT’; Right: *pdm*.); (**E**) The activity of ACX in the petals of ‘FT’ and *pdm* plants. (**F**,**G**) Western blot analysis of Bra040093-encoded protein expression. (Lanes 1–3: Petals of ‘FT’; lanes 4–6: Petals of *pdm*.).

In a previous study, using isolated microspore culture and ^60^Co γ-ray mutagenesis, we obtained a petal degeneration mutant (*pdm*) of the ‘FT’ line of Chinese cabbage (*Brassica campestris* ssp. *pekinensis*) [42]. Although, at the vegetative stage of growth, we detected no significant differences in the phenotypes of *pdm* and wild-type ‘FT’, at the reproductive growth stage the petals of *pdm* plants were observed to be degenerate, shriveled, incompletely expanded, and darker than those of ‘FT’ plants (Figure 1A–D). On the bases of the fine mapping of the mutant gene [43] and RNA sequencing (RNA-Seq) data [44], we predicted *Bra040093* to be a candidate gene associated with the *pdm* phenotype and gene annotation revealed that this gene encodes ACX1, which acts as a rate-limiting β-oxidation-related enzyme. In the present study, we further identified and analyzed the *pdm* candidate gene *Bra040093* in order to characterize the gene networks regulating petal development in *pdm* plants. The results lay the foundations for further elucidation of the molecular and regulatory mechanisms underlying floral organ development in Chinese cabbage.

## 2. Results

### 2.1. ACX Activity is Decreased in Pdm Plants

Analysis of ACX activity in the petals of ‘FT’ and *pdm* plants revealed that the activity of this enzyme was significantly lower in *pdm* plants than in ‘FT’ plants (Figure 1E).

### 2.2. Analysis of Protein Expression Patterns

In a previous study, we found that, compared with ‘FT’ plants, *Bra040093* was significantly down-regulated in *pdm* plants [43]. In the present study, we further analyzed the differential expression of the candidate gene *Bra040093* at the protein level by western blotting, using petals from ‘FT’ and *pdm* plants. We accordingly observed that expression of the Bra040093-encoded protein was significantly lower in the petals of *pdm* plants than in those of ‘FT’ plants, with the expression being barely detectable in the mutant (Figure 1F,G). These results were generally consistent with those obtained from the qRT-PCR analysis.

### 2.3. Analysis of JA and MeJ Contents

The RNA-Seq data indicated that the candidate gene, *Bra040093*, is involved in three β-oxidation reactions within the α-linolenic acid metabolic pathway, the end product of which is JA [44] (Figure S3.). We therefore measured the JA and MeJA contents in the petals of ‘FT’ and *pdm* plants and, accordingly, found that the contents of these two hormones were significantly lower in the petals of *pdm* plants than in those of ‘FT’ plants (Table 1).

### 2.4. Effects of Exogenous Application of JA and Meja on Petal Phenotype

**Figure 2:**
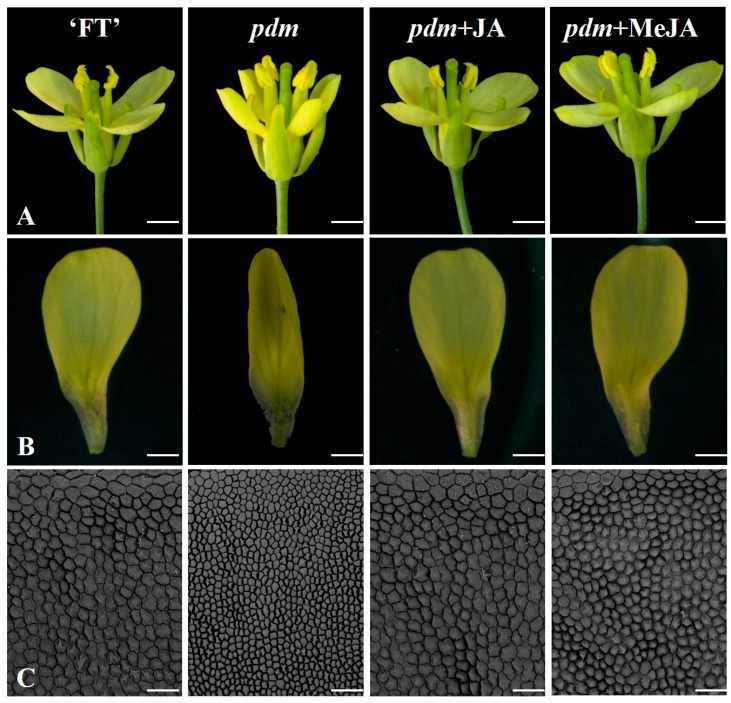
Phenotypic responses of ‘FT’ and *pdm* plants to exogenous treatments. (**A**) Flower, (**B**) petal, and (**C**) petal cells. (Scale bars: 20 μm).

**Figure 3:**
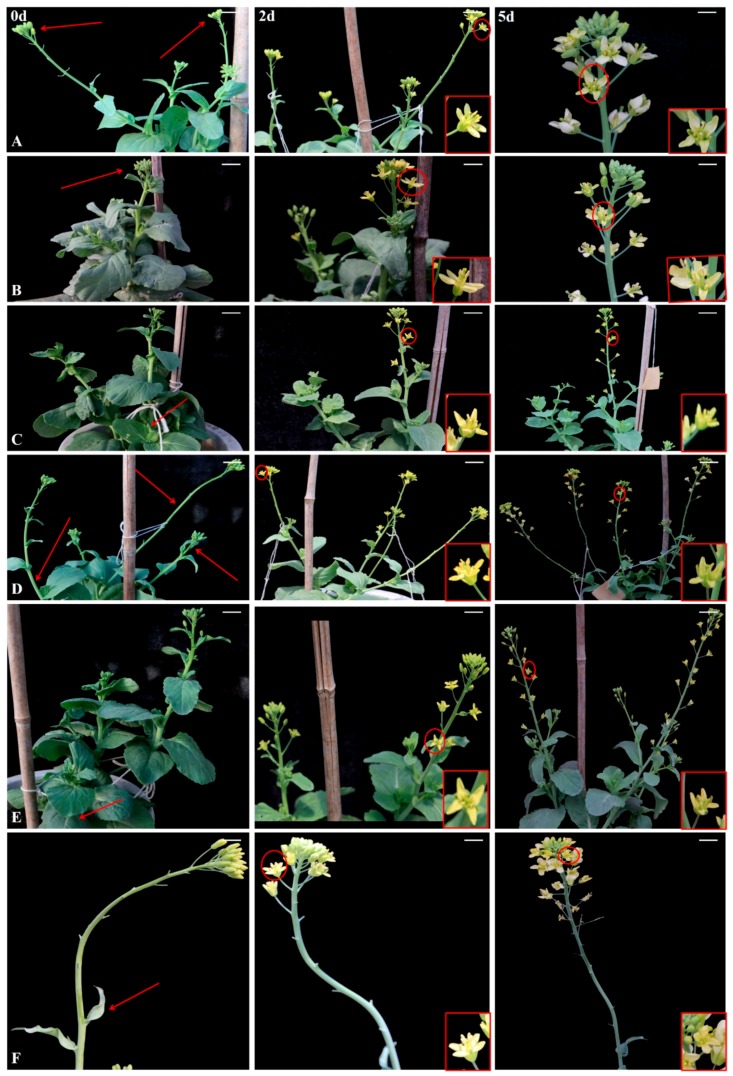
Effects of JA treatment on different tissues and parts of *pdm* plants. Phenotypes of (**A**) inflorescences, (**B**) petals, (**C**) vegetative stems. (**D**) Different parts of branches. (**E**) Basal leaves and (**F**) leaves on branches before (0 d) and after treatment (2 and 5 d) are shown. The arrows indicate the positions at which JA (1 mM) was applied.

On the basis of our observations of significant differences in the JA and MeJA contents of ‘FT’ and *pdm* plants, we examined the effects of exogenous application of JA and MeJA on the petal phenotype of *pdm* plants. As shown in Figure 2A,B, the petal phenotype of *pdm* returned to the wild-type phenotype, after 48 h, in response to exogenous JA application by spraying inflorescences and petals. However, on the fifth day, the effect of JA began to decline, and, in newly opened petals, the *pdm* phenotype was restored (Figure 3A,B). Similar results were obtained when inflorescences and petals were sprayed with MeJA (Figure 2A,B). As shown in Figure 2C, compared with ‘FT’ petals, the surface area of cells was decreased in *pdm* petals, whereas the surface area of these cells increased after exogenous application of JA, and the morphology and size of petals were similar to those of ‘FT’. These observations, accordingly, indicate that JA can regulate the size and shape of petals by influencing cell expansion.

To further study the effects of JA on *pdm* plants, in terms of the duration of the effects and the direction of hormone transport, different parts of the stem and leaves of different branches of *pdm* plants were treated with the JA solution. When the vegetative stems of *pdm* plants were sprayed with JA, the petal phenotype remained unchanged and was not restored to the normal ‘FT’ phenotype (Figure 3C). In contrast, whereas exogenous JA application to the lower parts of branches had no effect on the petal phenotype, when the upper and middle parts of branches were treated, the petal phenotype reverted to that of the wild type (Figure 3D). Moreover, JA treatment of basal leaves had no effect on the petal phenotype of *pdm* plants (Figure 3E), whereas treatment of the leaves on branches induced a normal phenotype in the petals of inflorescences positioned above the leaves but had no effect on petals of inflorescences positioned below the leaves (Figure 3F).

### 2.5. RNA-Seq Analysis

In order to examine the pathways via which JA may regulate petal development in *pdm* plants, we performed a comparative transcriptome analysis of ‘FT’, *pdm*, and pJA plants. We obtained a total of 45,254,135, 39,811,249, and 50,987,598 clean reads from ‘FT’, *pdm*, and pJA plants, respectively, of which 44,231,520 (87.12%), 38,821,450 (87.12%), and 49,835,756 (86.88%) reads were matched to unique genomic locations and could thus be used for gene expression analysis (Table S2).

**Figure 4:**
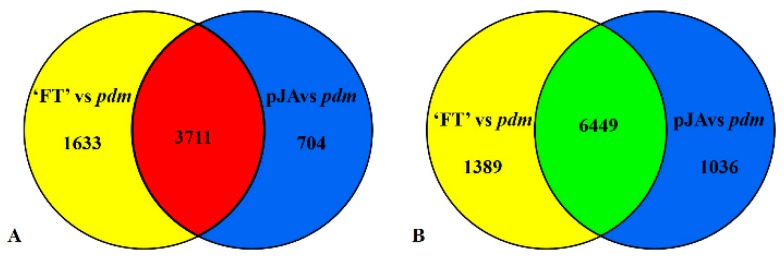
Numbers of differentially expressed genes (DEGs) identified in ‘FT’ vs. *pdm* and pJA vs. *pdm* comparisons. (**A**) Mutually up-regulated DEGs (red) and (**B**) mutually down-regulated DEGs (green).

We detected a total of 11,900 DEGs in the comparison between ‘FT’ and *pdm*, among which, 4415 genes were up-regulated and 7485 were down-regulated (Table S3). In the pJA vs. *pdm* comparison, a total of 13,182 DEGs were detected, of which 5344 were up-regulated and 7838 were down-regulated genes (Table S4). On the basis of these findings, we further examined those DEGs with consistent expressional tendencies and, accordingly, detected a total of 10,160 DEGs, among which there were 3711 genes that were upregulated and 6449 that were downregulated (Figure 4 and Table S5). These DEGs were mapped to 3856 GO terms using GO functional category analysis. GO analysis revealed a number of GO terms related to petal development, including response to hormone stimulus (64 DEGs), cellular response to hormone stimulus (2 DEGs), organ development (4 DEGs), tissue development (16 DEGs), cell development (14 DEGs), and regulation of cell development (1 DEG). As indicated, the response to hormone stimulus was the most significantly enriched GO term (Table S6). In addition, to determine the DEGs involved in metabolic pathways, a total of 123 KEGG pathways were mapped (Table S7). Of which, 15 pathways were significantly enriched, including those of α-linolenic acid metabolism (28 DEGs), plant hormone signal transduction (187 DEGs), and metabolic pathways (1034 DEGs). These results lay the foundation for further elucidating the mechanism whereby JA regulates petal development.

### 2.6. DEGs Related to Petal Development

The growth and development of floral organs involve the interplay of a complex array of physiological and biochemical processes associated with cell expansion and proliferation [45], controlled by the interaction of a large number of specific developmental regulatory genes. Among the 10,160 DEGs we detected that had consistent expression, we identified 69 genes related to floral organ development, including *Actin-depolymerizing factor 9* (*ADF9*: *Bra011575*), *SUPERMAN* (*SUP*: *Bra016878*), *WUSCHEL-related homeobox 3* (*WOX3*: *Bra001005* and *Bra039108*), *NAC domain-containing protein 100* (*NAC100*: *Bra012960*, *Bra002148*, *Bra023669*, *Bra009246*, and *Bra028685*), *ULTRAPETALA 1* (*ULT*: *Bra024219*), *ORGAN SIZE RELATED 1* (*ORS1*: *Bra004608* and *Bra016947*), *FLOWERING LOCUS T* (*FT*: *Bra022475*), and *EARLY FLOWERING 3* (*ELF*: *Bra031274*, *Bra001825*, and *Bra023887*) (Table S8). Among others, we further analyzed the following 11 genes involved in JA regulation of petal development: *APETALA1* (*AP1*: *Bra004361*), *v-myb avian myeloblastosis viral oncogene homolog* (*MYB21*: *Bra025300* and *Bra039067*), *JAGGED* (*JAG*: *Bra004312*, *Bra033931* and *Bra033930*), *SPATULA* (*SPT*: *Bra011742*), *TEOSINTE BRANCHED1*/*CYCLOIDEA*/*PCF4* (*TCP4*: *Bra021586*), *GRF-INTERACTING FACTOR1* (*GIF1*: *Bra020616* and *Bra036131*), and *SPIKE* (*SPK1*: *Bra038448*). Of these, *GIF1*, *JAG*, *AP1*, and *MYB21* were down-regulated and *SPIKE*, *SPT*, and *TCP4* were up-regulated in *pdm* plants, compared with wild-type ‘FT’ plants, with *SPT* (*Bra011742*) being specifically expressed in *pdm* and *JAG* (*Bra033931* and *Bra033930*) specifically expressed in ‘FT’ (Table 2).

### 2.7. qRT-PCR Analysis of Gene Expression Patterns

**Figure 5:**
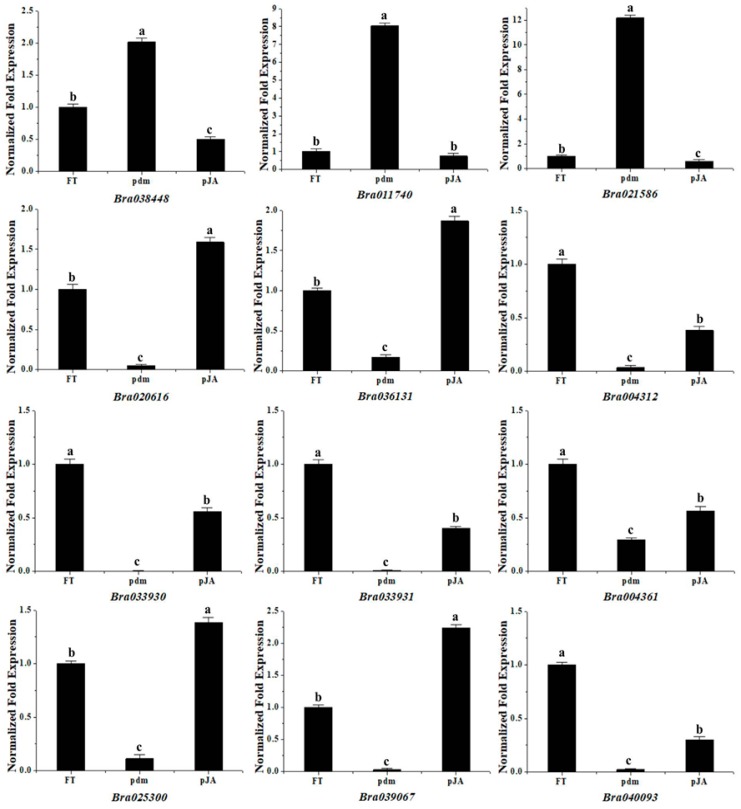
qRT-PCR analysis of gene expression patterns. Note: The relative expression levels of 12 differentially expressed genes.

To further examine the expression patterns of the detected DEGs, we selected 11 DEGs related to petal development and the mutant gene Bra040093 for qRT-PCR analysis using gene-specific primers. As shown in Figure 5, the expression patterns of these genes showed trends similar to those observed in the RNA-Seq data, thereby indicating that the transcriptome analysis was reliable.

## 3. Discussion

In previous studies, in which we fine-mapped mutated genes in the *pdm* mutant of Chinese cabbage, we identified *Bra040093* as the candidate gene for the degenerate petal trait and found that it encodes the oxidoreductase ACX1 [42,44]. Oxidoreductases like ACX act as peroxisomal flavoproteins [46] and the β-oxidation pathway in peroxisomes is known to play an important role in plant signal transduction and development, particularly with respect to JA biosynthesis and certain resistance reactions [47]. In tomato, a single point mutation of *LeACX1* resulted in the deficiency of JA production and compromised in its wound response [48]. Two acyl-CoA oxidases in soybean (*ACX1;1* and *ACX1;2*) had relatively stronger expression in the growing seedling axis and hypocotyl and weaker expression in the cotyledon [40]. Herbivore-induced acyl coenzyme gene *CsACX1* is involved in the synthesis of JA and expressed during the flowering in tea plants (*Camellia sinensis*) [41]. Moreover, in *Arabidopsis thaliana*, peroxisomal β-oxidation enzymes, such as *ACX*, 3-ketoacyl-CoA-thiolase, and multifunctional protein, have been shown to be required for inflorescence patterning [49,50,51]. To date, six ACX isozyme have been identified in *Arabidopsis thaliana* [52]. In this study, a small amount of ACX activity in the *pdm* plant may be related to isozymes.

Huang et al. [44] found that the candidate gene *Bra040093* is involved in α-linolenic acid metabolism (KO00592), which is one of the biosynthetic pathways of JA that plays a critical role in the development and differentiation of floral organs [23,45] and in which the *Bra040093* protein catalyzes the first β-oxidation reactions. Compared with the ‘FT’ wild-type Chinese cabbage, we found that *Bra040093* is down-regulated in *pdm* mutant plants and, accordingly, hypothesize that the *Bra040093* mutation in *pdm* might hinder JA biosynthesis and consequently affect petal development.

Previous studies have indicated that JA may be an important regulatory factor in the process of petal development and petal identification genes have been shown to be related to JA biosynthesis at the stage of petal development [13]. In the JA biosynthetic pathway of *Arabidopsis thaliana*, *BPEp* acts downstream of *OPR3* and the *opr3-bpe-1* double mutant produces large petals. However, this phenotype was found to revert back to the wild-type petal phenotype in response to exogenous application of MeJA [14]. Inspired by these findings, and to further verify our aforementioned hypothesis, in the present study we sought to determine JA and MeJA contents in the petals of *pdm* plants and, accordingly, found that the levels of these two hormones were significantly lower in the *pdm* mutant than in the ‘FT’ wild type. Thus, we next examined the effects of exogenous application of JA and MeJA on the *pdm* mutant and found that the petal phenotype of *pdm* returned to the wild-type phenotype in response to spraying with these two hormones. Thus, these results indicate that JA is relevant to petal development in Chinese cabbage and indirectly verify the function of the candidate gene *Bra040093*. The mutant gene *Bra040093* of *pdm* is involved in JA biosynthesis. JA is a plant hormone with a wide range of physiological effects and is involved in plant responses to biotic and abiotic stresses [19,20,21,22,23]. Therefore, the mutant *pdm* may be an ideal material for studying the resistance in Chinese cabbage.

The duration of the effects of JA and the direction of its transport were also investigated by spraying different parts of *pdm* plants with the JA solution and monitoring the responses of the inflorescences. The effects of JA generally became evident on the second day (48 h) after commencing treatment; however, they tended to wane after the fifth day. When stems and leaves were sprayed, we observed that the effect of JA was dependent on transport distance, with the effect tending to decrease and eventually become undetectable with increasing transport distance. Moreover, we demonstrated that JA may transport in an upward direction. These findings provide an important basis for further studies aimed at elucidating the mode of action of JA in the regulation of petal development.

**Figure 6:**
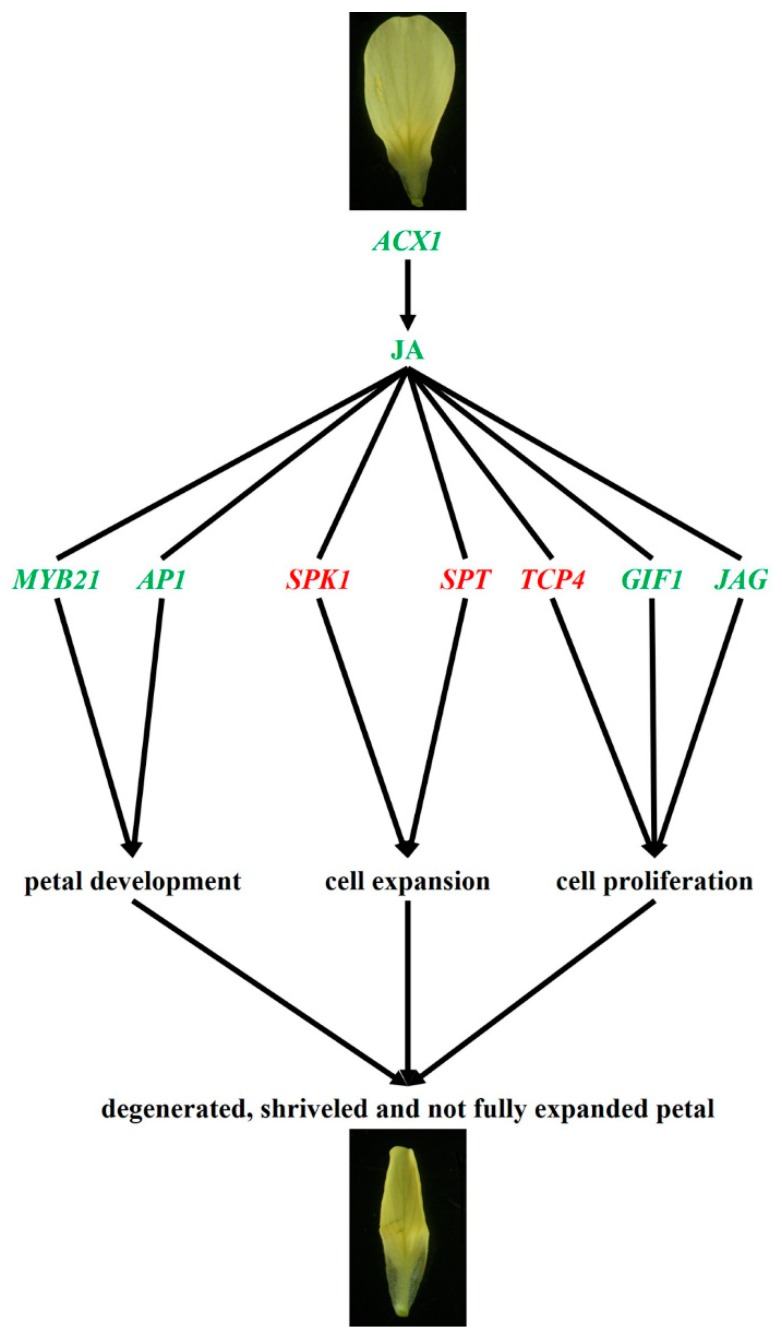
Proposed JA regulatory pathways that contribute to petal development in *pdm* plants. Note: Genes indicated by red text are up-regulated and those indicated by green are down-regulated.

In order to study the mechanisms whereby JA regulates petal development, we undertook comparative transcriptome analyses of ‘FT’, *pdm*, and pJA plants. By determining the DEGs with consistent expressional tendencies in our comparisons of ‘FT’ and *pdm* plants and pJA and *pdm* plants, we were able to identify those genes related to petal development, which may be involved in the JA-mediated regulation of petal development. Among the genes thus identified, it has previously been shown that *MYB21* promotes the development of stamens and petals downstream of the JA signal [34,35]. Furthermore, in the ABC model of floral organ development, *AP1*, another of the genes we identified, is a Class A gene in A. thaliana, which determines the development of petals and sepals. Knockout of the *AP1* gene in *Arabidopsis thaliana* can transform the sepals into leaves and bract structures, even leading to petal deficiency [3,53]. *SPK1* encodes a *guanine nucleotide exchange factor* (*GEF*) that affects the anisotropic growth of petals [54,55] and it has been found that petals of an *SPK1* knockdown mutant were significantly longer and narrower than those of the wild type [56]. *SPT* encodes a *bHLH* transcription factor that is involved in the inhibition of cotyledon, leaf, and petal expansion [57] and, compared with the wild type, both *spt-2* and *spt-10* mutants were found to have increased petal expansion [58,59]. Members of the *TCP* transcription factor family can regulate the development of plant organs and, in *Arabidopsis thaliana*, over-expression of *TCP4* can lead to smaller flower organs during the process of flower development [60]. *GIF* proteins, including *GIF1*, *GIF2*, and *GIF3*, are a class of co-transcriptional activity factors [61] that play a positive role in regulating cell proliferation. The *Arabidopsis gif1* mutant was found to have petals that were narrower and smaller in area, width, and length, compared with Col wild-type plants [62]. *JAG* encodes a transcription factor with a single *C_2_H_2_* zinc-finger domain and functions as a direct mediator between genes, thereby controlling the identity of organs and tissues [63]. Compared to the wild type, the sepals and petals of the *jag-1* mutant were observed to be shorter and narrower [64]. Our results, in the present study, indicate that, compared with the wild-type ‘FT’ plants, *Bra040093* is down-regulated in *pdm* plants, leading to a decrease in the JA content of these plants. Altered JA content may, in turn, induce corresponding changes in the genes that respond to JA signaling [18]. In this study, we identified a total of seven regulatory factors that are responsive to JA, namely, *MYB21*, *AP1*, *SPK1*, *SPT*, *TCP4*, *GIF1*, and *JAG*, among which, *MYB21* and *AP1* can directly regulate petal development, *SPK1* and *SPT* can regulate petal morphology through cell expansion, and *TCP4*, *GIF1*, and *JAG* can regulate petal morphology via cell proliferation. Ultimately, the combined regulatory effects of these genes may result in the degenerate, shriveled, and incompletely expanded petals observed in *pdm* plants (Figure 6).

## 4. Materials and Methods

### 4.1. Plant Material and Growth Conditions

The *pdm* plants used in the present study were derived from the ‘FT’ doubled haploid line of Chinese cabbage using a combination of isolated microspore culture and radiation mutagenesis (^60^Co-γ rays) [42]. Mutant *pdm* plants are diploid and flow cytometry analysis has confirmed that the mutation is stably inherited over multiple generations.

In January 2017, germinated seeds of ‘FT’ and *pdm* were vernalized for 15 days and sown in a greenhouse at Shenyang Agricultural University, Liaoning Sheng, China. In March 2017, plants at the full-bloom stage were selected for the experiments described below.

### 4.2. Measurement of ACX Activity

The activity of ACX in the petal of ‘FT’ and *pdm* plants was measured according to the modified method described by Adham et al. [65] and Liu et al. [66]. Each 70 µL reaction consisted of 1 µL of (0.8 mM) fatty acyl-CoA substrate solution (Sigma-Aldrich, St. Louis, MO, USA), 35 µL of color substrate solution (50 mM p-hydroxybenzoic acid, 110 U horseradish peroxidase, and 2 mM 4-aminoantipyrine), and 34 µL of crude enzyme extract. As an indirect measure of ACX activity, we determined hydrogen peroxide (H_2_O_2_) production using an Infinite M200 PRO microplate reader (Tecan, Zurich, Switzerland) at 500 nm. A standard curve of H_2_O_2_ was used to determine H_2_O_2_ concentrations (Figure S1) and to calculate reaction rates (pmol H_2_O_2_ mg^−1^ min^−1^). Measurements were obtained from three independent biological replicates and data were analyzed using Origin Pro 8.0 software (https://www.originlab.com/) (access on 28th June 2017) and Data Processing System (*p* < 0.05).

### 4.3. Western Blotting

On the basis of the amino acid sequence of the candidate gene *Bra040093* in ‘FT’ plants, the N-terminal 465–665 amino acids were selected as an immunogen. A polyclonal antibody to this sequence was synthesized by ProbeGene (Jiangsu, China) and this was used as a primary antibody (1:500 dilution) for western blotting. As a primary antibody for the Actin reference gene, we used an anti-plant actin mouse mAb (3T3) (Abbkine, Wuhan, China, 1:5000 dilution). Total proteins were extracted from different tissues using a Total Protein Extraction Kit (Invent Biotechnologies, Plymouth, MN, USA), and protein concentration was measured using a 2-D Quant Kit (GE Healthcare, Ltd., Little Chalfont, UK). A total of 100 μg of total protein was loaded per lane. Proteins were resolved by sodium dodecyl sulphate polyacrylamide gel electrophoresis (12% gel) and transferred to a polyvinylidene difluoride (PVDF) membrane (Millipore, Burlington, MA, USA) using a Trans-Blot Turbo transfer system (Bio-Rad Laboratories, Hercules, CA, USA). PVDF membranes were blocked in 5% bovine serum albumin (TIANDZ, Beijing, China) at 37 °C for 2 h, incubated overnight with the primary antibody (1:500) at 4 °C, and the following day incubated with the secondary antibody (peroxidase-conjugated AffiniPure goat anti-rabbit IgG; ZSGB-BIO, Beijing, China) (1:5000) at 37 °C for 1 h. Thereafter, the membranes were developed in the dark for 2 to 3 min using Pro-light HRP chemiluminescence detection reagent (TIANGEN Biotech, Beijing, China). Images were obtained using a Tanon 5200 chemiluminescence imaging system (Tanon Science & Technology Co., Ltd., Shanghai, China).

### 4.4. Measurement of JA and MeJA Contents

The JA and MeJA contents of the petals of ‘FT’ and *pdm* plants were measured according to the ESI-HPLC-MS/MS method described by You et al. [67] and Liu et al. [68]. Tissue samples (0.5 g) were ground to a powder using liquid nitrogen. After adding 5 mL isopropanol/hydrochloric acid extraction buffer to the powder, the mixture was shaken at 4 °C for 30 min. Thereafter, dichloromethane was added and the mixture was shaken at 4 °C for a further 30 min. The sample was then centrifuged at 12,000× *g* for 5 min. The resulting lower organic phase was maintained in the dark, whereas the upper organic phase was blow-dried using a pressure blowing concentrator (Miulab, Hangzhou, China) and dissolved in 400 µL of methanol (containing 0.1% formic acid). The solution thus obtained was filtered through a 0.22 µm nylon membrane and the filtrate was collected for examination.

The contents of JA and MeJA in each sample were determined by high-performance liquid chromatography (Agilent Technology, Palo Alto, CA, USA) under liquid phase conditions. The chromatographic column used was a Poroshell 120 SB-C18 (2.1 × 150, 2.7 µm), the column temperature was 30 °C, the mobile phase was A:B = (methanol/0.1% formic acid):(water/0.1% formic acid), the flow rate was 0.3 mL/min, and the injection volume was 2 µL. Quantitative analysis was conducted using the external standard method with JA standard samples of 0.2, 0.5, 1, 2, 5, 10, 20, 50, and 200 ng/mL (Figure S2). Measurements were obtained from three independent biological replicates and the data were analyzed using SPSS 16.0 (spss inc., Chicago, IL, USA).

### 4.5. Exogenous Application of JA and MeJA

At the full-bloom stage, robust and phenotypically similar ‘FT’ and *pdm* plants were selected as experimental materials. During the evening period (16:00–18:00), all blooming flowers in the inflorescences were removed before subjecting the plants to hormone treatment.

Different tissues and positions of *pdm* plants were evenly sprayed with 2 mL JA (1 mM, in 1% ethanol (v/v) in water) or 2 mL MeJA (0.1 mM, in 1% ethanol (v/v) in water) per plant for one treatment. As controls, we sprayed plants with 1% ethanol (v/v). Parts of the plants that needed to be shielded from treatment were wrapped in preservative film, which was removed after 12 h, and phenotypic changes were subsequently examined.

### 4.6. Scanning Electron Microscopy (SEM)

The petals of the ‘FT’, *pdm*, *pdm* + JA and *pdm* + MeJA plants were fixed in a solution of FAA (100 mL: 89 mL 50% ethanol, 6 mL glacial acetic acid, 5 mL formaldehyde) at 4 ℃ for 12 h. The fixed samples were then dehydrated using gradient series of 50%, 75%, 90%, and 100% ethanol (each for 3 min) and then with 50% ethanol + 50% tert-butanol, 25% ethanol + 75% tert-butanol, 10% ethanol + 90% tert-butanol, and 100% tert-butanol (each for 3 min). Thereafter, the samples were immersed in 100% tert-butanol and desiccated in an ES-2030 lyophilizer (Hitachi, Tokyo, Japan). The dried samples were adhered to a sample platform using conductive adhesive and were gold-palladium sputter-coated using an MSP-2S Carbon coater (Hitachi, Tokyo, Japan). Finally, the samples were observed and photographed using a TM3030 scanning electron microscope (Hitachi, Tokyo, Japan).

### 4.7. RNA-Seq, Expression Annotation, GO, and KEGG Pathway Enrichment Analyses

After trimming the adapter sequences from the reads, we removed poly-N sequences and low-quality reads (Q ≤ 20) to give high-quality clean reads. These reads were subsequently mapped to the brassica reference genome (http://brassicadb.org) using HISAT software.

DESeq software and FPKM values (expected number of fragments per kilobase of transcript sequence per million base pairs sequenced) were used to estimate the gene expression level of each sample. In this study, we defined differentially expressed genes (DEGs) as those showing |log2(FoldChange)| > 1 and padj < 0.05.

In order to further study the biological function of DEGs and the associated metabolic pathways, we performed GO functional and KEGG pathway enrichment analyses. The significantly enriched GO terms and KEGG pathways of DEGs were determined by comparison with the genome background using hypergeometric tests, with a corrected *p*-value < 0.05 used as the threshold.

### 4.8. RNA Isolation, cDNA Library Construction, and Illumina Sequencing for Transcriptome Analysis

Petals were selected from the 10 phenotypically similar plants of ‘FT’, *pdm*, and *pdm* + JA, respectively. The extraction of total RNA and cDNA synthesis were respectively conducted using an RNApure Total RNA Kit (Aidlab, Beijing, China) and a HiScript II Q Select RT SuperMix for qPCR (Vazyme, Nanjing, China) according to the manufacturer’s instructions. Equal amounts of total RNA from three biological replicates of ‘FT’, *pdm*, and *pdm* + JA were respectively pooled for RNA-Seq library construction, which we designated as FT-1, FT-2, and FT-3; pdm-1, pdm-2, and pdm-3; and pJA-1, pJA-2, and pJA-3.

The quantity and purity of total RNA were determined using a Bioanalyzer 2100 and RNA 6000 Nano LabChip Kit (Agilent, Carpinteria, CA, US) with RIN number > 7.0. The nine cDNA libraries were sequenced with 6 G depth using the Hiseq4000-PE150 sequencing platform (Illumina, San Diego, CA, USA) by Novogene (Beijing, China).

### 4.9. Quantitative Real-Time PCR (qRT-PCR) Analysis

The cDNAs obtained by reverse transcription from the petals of ‘FT’, *pdm*, and pJA plants were used as templates for qRT-PCR using UltraSYBR Mixture (CWBIO) in a QuantStudio 6 Flex Real-Time PCR System (Applied Biosystems, Foster City, CA, USA), according to the manufacturer’s instructions. The gene-specific primers were designed using Primer Premier 5.0 software, based on the reference sequences (http://brassicadb.org/brad/) (access on 28th October 2018), and the Actin gene was used as the internal control (Table S1). All reactions were conducted in triplicate using three independent biological replicates. The data were analyzed using OriginPro8.0 software.

## 5. Conclusions

The development of floral organs is a complex physiological and biochemical process, involving the regulation of floral organ size via changes in cell proliferation and growth [69] and the integration and coordination of hormone signaling pathways in the regulatory network [70,71,72,73]. In the present study, we indirectly verified the function of the *Bra040093* gene. Compared with the wild-type ‘FT’ Chinese cabbage, mutation of *Bra040093* in *pdm* was found to lead to reduced JA levels, thereby influencing petal development. On the basis of these results, we conducted comparative transcriptome analyses to further investigate the regulatory effect of JA on petal development and, accordingly, detected a number of DEGs related to petal development, the mutual regulation of which may eventually lead to the degenerate petal phenotype of *pdm* plants. Our findings not only contribute to revealing the molecular mechanisms of petal development in Chinese cabbage but also lay theoretical foundations for intensive studies on the mechanisms underlying floral organ development in plants.

## Figures and Tables

**Table 1:** JA and MeJA contents in petals of ‘FT’ and *pdm* plants.

Materials	JA (ng/g)	MeJA (ng/g)
‘FT’	43.07 ± 1.92 *	1.4 ± 0.06 *
*pdm*	1.76 ± 0.06	0.28 ± 0.02

Note: Data are the means and standard deviation (SD) from three independent replicates. * *p* < 0.05, *t*-test.

**Table 2:** The differentially expressed genes associated with petal development (pdm vs. ‘FT’).

Gene ID	log_2_ Fold Change	Up/Down Regulation	Gene Annotation (BLASTX to *Arabidopsis thaliana*)
*Bra038448*	1.63	Up	*Guanine nucleotide exchange factor SPIKE 1* (*SPK1*)
*Bra011740*	3.93	Up	*Transcription factor SPATULA* (*SPT*)
*Bra021586*	4.47	Up	*Transcription factor TCP4* (*TCP4*)
*Bra020616*	−2.43	Down	*GRF1-interacting factor 1* (*GIF1*)
*Bra036131*	−3.20	Down	*GRF1-interacting factor 1* (*GIF1*)
*Bra004312*	−3.01	Down	*Zinc finger protein JAGGED* (*JAG*)
*Bra033931*	−4.65	Down	*Zinc finger protein JAGGED* (*JAG*)
*Bra033930*	−4.39	Down	*Zinc finger protein JAGGED* (*JAG*)
*Bra004361*	−1.10	Down	*Floral homeotic protein APETALA 1* (*AP1*)
*Bra025300*	−2.38	Down	*Transcription factor MYB21* (*MYB21*)
*Bra039067*	−4.21	Down	*Transcription factor MYB21* (*MYB21*)

## Supplementary Materials

Supplementary materials can be found at https://www.mdpi.com/1422-0067/20/9/2310/s1.

## Database

All raw sequence data have been deposited in NCBI Sequence Read Archive (https://submit.ncbi.nlm.nih.gov/subs/sra/) (access on 19th October 2018). Under the BioProject ID: PRJNA497343.

## References

[1] Crepet W.L. (2000). Progress in understanding angiosperm history, success, and relationships: Darwin’s abominably “perplexing phenomenon”. Proc. Natl. Acad. Sci. USA.

[2] Zhang X., Zhou Y., Ding L., Wu Z., Liu R., Meyerowitz E.M. (2013). Transcription repressor HANABA TARANU controls flower development by integrating the actions of multiple hormones, floral organ specification genes, and GATA3 family genes in *Arabidopsis*. Plant Cell.

[3] Coen E.S., Meyerowitz E.M. (1991). The war of the whorls: Genetic interactions controlling flower development. Nature.

[4] Colombo L., Franken J., Koetje E., van Went J., Dons H.J., Angenent G.C., van Tunen A.J. (1995). The petunia MADS box gene *FBP11* determines ovule identity. Plant Cell.

[5] Theissen G. (2001). Development of floral organ identity: Stories from the MADS house. Curr. Opin. Plant Biol..

[6] Theissen G., Saedler H. (2011). Plant biology. Floral quartets. Nature.

[7] Galliot C., Stuurman J., Kuhlemeier C. (2006). The genetic dissection of floral pollination syndromes. Curr. Opin. Plant Biol..

[8] Mizukami Y., Fischer R.L. (2000). Plant organ size control: *AINTEGUMENTA* regulates growth and cell numbers during organogenesis. Proc. Natl. Acad. Sci. USA.

[9] Anastasiou E., Kenz S., Gerstung M., MacLean D., Timmer J., Fleck C., Lenhard M. (2007). Control of plant organ size by *KLUH/CYP78A5*-Dependent intercellular signaling. Dev. Cell.

[10] Stransfeld L., Eriksson S., Adamski N.M., Breuninger H., Lenhard M. (2010). *KLUH/CYP78A5* promotes organ growth without affecting the size of the early primordium. Plant Signal Behav..

[11] Kawade K. (2015). Proliferative control of leaf cells through inter-cell-layer AN3 signaling. Plant Morphol..

[12] Adamski N.M., Anastasiou E., Eriksson S., O’Neill C.M., Lenhard M. (2009). Local maternal control of seed size by *KLUH/CYP78A5*-dependent growth signaling. Proc. Natl. Acad. Sci. USA.

[13] Brioudes F., Joly C., Szécsi J., Varaud E., Leroux J., Bellvert F., Bertrand C., Bendahmane M. (2009). Jasmonate controls late development stages of petal growth in *Arabidopsis thaliana*. Plant J..

[14] Szécsi J., Joly C., Bordji K., Varaud E., Cock J.M., Dumas C., Bendahmane M. (2006). *BIGPETALp*, a *bHLH* transcription factor is involved in the control of *Arabidopsis* petal size. EMBO J..

[15] Varaud E., Brioudes F., Szécsi J., Leroux J., Brown S., Perrot-Rechenmann C., Bendahmane M. (2011). AUXIN RESPONSE FACTOR8 regulates *Arabidopsis* petal growth by interacting with the bHLH transcription factor BIGPETALp. Plant Cell.

[16] Szécsi J., Wippermann B., Bendahmane M. (2014). Genetic and phenotypic analyses of petal development in *Arabidopsis*. Methods Mol. Biol..

[17] Song S., Qi T., Wasternack C., Xie D. (2014). Jasmonate signaling and crosstalk with gibberellin and ethylene. Curr. Opin. Plant Biol..

[18] Yuan Z., Zhang D. (2015). Roles of jasmonate signalling in plant inflorescence and flower development. Curr. Opin. Plant Biol..

[19] Yoon J.Y., Hamayun M., Lee S.K., Lee I.J. (2009). Methyl jasmonate alleviated salinity stress in soybean. J. Crop Sci. Biotechnol..

[20] Wasternack C., Stenzel I., Hause B., Hause G., Kutter C., Maucher H., Neumerkel J., Feussner I., Miersch O. (2006). The wound response in tomato-role of jasmonic acid. J. Plant Physiol..

[21] Fugate K.K., Oliveira L.S.D., Ferrareze J.P., Bolton M.D., Deckard E.L., Finger F.L. (2017). Jasmonic acid causes short and long term alterations to the transcriptome and the expression of defense genes in sugarbeet roots. Plant Gene.

[22] He Y., Zhang H., Sun Z., Li J., Hong G., Zhu Q., Zhou X., MacFarlane S., Yan F., Chen J. (2017). Jasmonic acid-mediated defense suppresses brassinosteroid-mediated susceptibility to Rice black streaked dwarf virus infection in rice. New Phytol..

[23] Wasternack C., Hause B. (2013). Jasmonates: Biosynthesis, perception, signal transduction and action in plant stress response, growth and development. an update to the 2007 review in Annals of Botany. Ann. Bot..

[24] Monzón G.C., Pinedo M., Lamattina L., Canal L.D.L. (2012). Sunflower root growth regulation: The role of jasmonic acid and its relation with auxins. Plant Growth Regul..

[25] Stintzi A., Browse J. (2000). The *Arabidopsis* male-sterile mutant, *opr3*, lacks the 12-oxophytodienoic acid reductase required for jasmonate synthesis. Proc. Natl. Acad. Sci. USA.

[26] Li L., Zhao Y., McCaig B.C., Wingerd B.A., Wang J., Whalon M.E., Pichersky E., Howe G.A. (2004). The tomato homolog of coronatine-insensitive1 is required for the maternal control of seed maturation, jasmonate-signaled defense responses, and glandular trichome development. Plant Cell.

[27] Ishiguro S., Kawai-Oda A., Ueda J., Nishida I., Okada K. (2001). The *DEFECTIVE IN ANTHER DEHISCEBCE1* gene encodes a novel phospholipase A1 catalyzing the initial step of jasmonic acid biosynthesis, which synchronizes pollen maturation, anther dehiscence, and flower opening in Arabidopsis. Plant Cell.

[28] Reeves P.H., Ellis C.M., Ploense S.E., Wu M.F., Yadav V., Tholl D., Chételat A., Haupt I., Kennerley B.J., Hodgens C. (2012). A regulatory network for coordinated flower maturation. PLoS Genet..

[29] Ueda J., Kato J. (1980). Isolation and identification of a senescence-promoting substance from wormwood (*Artemisia absinthium* L.). Plant Physiol..

[30] Liu L., Li H.X., Zeng H.L., Cai Q.H., Zhou X., Yin C.X. (2016). Exogenous jasmonic acid and cytokinin antagonistically regulate rice flag leaf senescence by mediating chlorophyll degradation, membrane deterioration, and senescence-associated genes expression. Plant Growth Regul..

[31] Li H., Xue D., Gao Z., Yan M., Xu W., Xing Z., Huang D., Qian Q., Xue Y. (2009). A putative lipase gene *EXTRA GLUME1* regulates both empty-glume fate and spikelet development in rice. Plant J..

[32] Cai Q., Yuan Z., Chen M., Yin C., Luo Z., Zhao X., Liang W., Hu J., Zhang D. (2014). Jasmonic acid regulates spikelet development in rice. Nat. Commun..

[33] Pak H., Guo Y., Chen M., Chen K., Li Y., Hua S., Shamsi I., Meng H., Shi C., Jiang L. (2009). The effect of exogenous methyl jasmonate on the flowering time, floral organ morphology, and transcript levels of a group of genes implicated in the development of oilseed rape flowers (*Brassica napus* L.). Planta.

[34] Mandaokar A., Thines B., Shin B., Lange B.M., Choi G., Koo Y.J., Yoo Y.J., Choi Y.D., Choi G., Browse J. (2006). Transcriptional regulators of stamen development in Arabidopsis identified by transcriptional profiling. Plant J..

[35] Cheng H., Song S., Xiao L., Soo H.M., Cheng Z., Xie D., Peng J. (2009). Gibberellin acts through jasmonate to control the expression of *MYB21*, *MYB24*, and *MYB57* to promote stamen filament growth in *Arabidopsis*. PLoS Genet..

[36] Song S., Qi T., Huang H., Ren Q., Wu D., Chang C., Peng W., Liu Y., Peng J., Xie D. (2011). The Jasmonate-ZIM Domain Proteins Interact with the R2R3-MYB Transcription Factors MYB21 and MYB24 to Affect Jasmonate-Regulated Stamen Development in *Arabidopsis*. Plant Cell.

[37] León J. (2013). Role of plant peroxisomes in the production of jasmonic acid-based signals. Subcell. Biochem..

[38] Li C., Schilmiller A.L., Liu G., Lee G.I., Howe G.A. (2005). Role of -oxidation in jasmonate biosynthesis and systemic wound signaling in tomato. Plant Cell.

[39] Schilmiller A.L., Howe K.G.A. (2007). Functional diversification of acyl-coenzyme a oxidases in jasmonic acid biosynthesis and action. Plant Physiol..

[40] Agarwal A.K., Qi Y., Bhat D.G., Woerner B.M., Brown S.M. (2001). Gene isolation and characterization of two acyl coa oxidases from soybean with broad substrate specificities and enhanced expression in the growing seedling axis. Plant Mol. Biol..

[41] Xin Z.J., Chen S.L., Ge L.G., Li X.W., Sun X.L. (2019). The involvement of a herbivore-induced acyl-CoA oxidase gene, *CsACX1*, in the synthesis of jasmonic acid and its expression in flower opening in tea plant (*Camellia sinensis*). Plant Physiol. Biochem..

[42] Huang S.N., Liu Z.Y., Li D.Y., Yao R.P., Meng Q., Feng H. (2014). Screening of Chinese cabbage mutants produced by ^60^Coγ-ray mutagenesis of isolated microspore cultures. Plant Breed..

[43] Huang S.N., Liu Z.Y., Yao R.P., Li D.Y., Zhang T., Li X., Hou L., Wang Y.H., Tang X.Y., Feng H. (2016). Candidate gene prediction for a petal degeneration mutant, *pdm*, of the Chinese cabbage (*Brassica campestris*, ssp. *pekinensis*) by using fine mapping and transcriptome analysis. Mol. Breed..

[44] Huang S.N., Liu Z.Y., Yao R.P., Li D.Y., Feng H. (2015). Comparative transcriptome analysis of the petal degeneration mutant *pdm*, in Chinese cabbage (*Brassica campestris*, ssp. *pekinensis*) using RNA-seq. Mol. Genet. Genom..

[45] Horiguchi G., Ferjani A., Fujikura U., Tsukaya H. (2006). Coordination of cell proliferation and cell expansion in the control of leaf size in *Arabidopsis thaliana*. J. Plant Res..

[46] Lazarow P.B., De Duve C. (1976). A fatty acyl-CoA oxidizing system in rat liver peroxisomes; enhancement by clofibrate, a hypolipidemic drug. Proc. Natl. Acad. Sci. USA.

[47] Kondo S., Setha S., Rudell D.R., Buchanan D.A., Mattheis J.P. (2005). Aroma volatile biosynthesis in apples affected by 1-MCP and methyl jasmonate. Postharvest Biol. Technol..

[48] Powers R.A., Rife C.L., Schilmiller A.L., Howe G.A., Garavito R.M. (2006). Structure determination and analysis of acyl-coa oxidase (ACX1) from tomato. Acta Crystallogr..

[49] Wasternack C. (2007). Jasmonates: An update on biosynthesis, signal transduction and action in plant stress response, growth and development. Ann. Bot..

[50] Koo A.J., Howe G.A. (2012). Catabolism and deactivation of the lipid-derived hormone jasmonoyl-isoleucine. Front. Plant Sci..

[51] Wiszniewski A.A., Bussell J.D., Long R.L., Smith S.M. (2014). Knockout of the two evolutionarily conserved peroxisomal 3-ketoa cyl-CoA thiolases in *Arabidopsis* recapitulates the *abnormal inflorescence meristem 1* phenotype. J. Exp. Bot..

[52] Hooks M.A., Kellas F., Graham I.A. (1999). Long-chain acyl-CoA oxidases of *Arabidopsis*. Plant J..

[53] Gustafson-Brown C., Savidge B., Yanofsky M.F. (1994). Regulation of the *Arabidopsis* floral homeotic gene *APETALA1*. Cell.

[54] Basu D., Le J., Zakharova T., Mallery E.L., Szymanski D.B. (2008). A SPIKE1 signaling complex controls actin-dependent cell morphogenesis through the heteromeric WAVE and APR2/3 complexes. Proc. Natl. Acad. Sci. USA.

[55] Qiu J.L., Jilk R., Marks M.D., Szymanski D.B. (2002). The Arabidopsis *SPIKE1* gene is required for normal cell shape control and tissue development. Plant Cell.

[56] Ren H., Dang X., Yang Y., Huang D., Liu M., Gao X., Lin D. (2016). SPIKE1 Activates ROP GTPase to Modulate Petal Growth and Shape. Plant Physiol..

[57] Groszmann M., Bylstra Y., Lampugnani E.R., Smyth D.R. (2010). Regulation of tissue-specific expression of *SPATULA*, a bHLH gene involved in carpel development, seedling germination, and lateral organ growth in *Arabidopsis*. J. Exp. Bot..

[58] Heisler M.G., Atkinson A., Bylstra Y.H., Walsh R., Smyth D.R. (2001). *SPATULA*, a gene that controls development of carpel margin tissues in *Arabidopsis*, encodes a bHLH protein. Development.

[59] Penfield S., Josse E.M., Kannangara R., Gilday A.D., Halliday K.J., Graham I.A. (2005). Cold and Light Control Seed Germination through the bHLH Transcription Factor SPATULA. Curr. Biol..

[60] Nag A., King S., Jack T. (2009). miR319a targeting of *TCP4* is critical for petal growth and development in *Arabidopsis*. Proc. Natl. Acad. Sci. USA.

[61] Lee B.H., Ko J.H., Lee S., Lee Y., Pak J.H., Kim J.H. (2009). The Arabidopsis *GRF-INTERACTING FACTOR* gene family performs an overlapping function in determining organ size as well as multiple developmental properties. Plant Physiol..

[62] Kim J.H., Kende H. (2004). A transcriptional coactivator, AtGIF1, is involved in regulating leaf growth and morphology in *Arabidopsis*. Proc. Natl. Acad. Sci. USA.

[63] Dinneny J.R., Yadegari R., Fischer R.L., Yanofsky M.F., Weigel D. (2004). The role of *JAGGED* in shaping lateral organs. Development.

[64] Schiessl K., Muiño J.M., Sablowshi R. (2014). *Arabidopsis JAGGED* links floral organ patterning to tissue growth by repressing Kip-related cell cycle. Proc. Natl. Acad. Sci. USA.

[65] Adham A.R., Zolman B.K., Millius A., Bartel B. (2005). Mutations in Arabidopsis acyl-CoA oxidase genes reveal distinct and overlapping roles in beta-oxidation. Plant J..

[66] Liu W.L., Jia H.J., Zhang X. (2010). Determination of acyl Coenzyme a Oxidase activity in Peach Fruit. Zhejiang Agric. Sci..

[67] You C.C., Zhu H.L., Xu B.B., Huang W.X., Wang S.H., Ding Y.F., Liu Z.H., Li G.H., Lin C., Ding C.Q. (2016). Effect of removing superior spikelets on grain filling of inferior spikelets in rice. Front. Plant Sci..

[68] Liu T., Xu J., Li J., Hu X. (2018). No is involved in JA- and H_2_O_2_-mediated ala-induced oxidative stress tolerance at low temperatures in tomato. Environ. Exp. Bot..

[69] Krizek B.A., Anderson J.T. (2013). Control of flower size. J. Exp. Bot..

[70] Pelaz S., Ditta G.S., Baumann E., Wisman E., Yanofsky M.F. (2000). B and C floral organ identity functions require sepallata MADS-box genes. Nature.

[71] Hu Y., Poh H.M., Chua N.H. (2006). The Arabidopsis *ARGOS-LIKE* gene regulates cell expansion during organ growth. Plant J..

[72] Fujikura U., Horiguchi G., Ponce M.R., Micol J.L., Tsukaya H. (2009). Coordination of cell proliferation and cell expansion mediated by ribosome-related processes in the leaves of *Arabidopsis thaliana*. Plant J..

[73] Feng G., Qin Z., Yan J., Zhang X., Hu Y. (2011). Arabidopsis *ORGAN SIZE RELATED1* regulates organ growth and final organ size in orchestration with *ARGOS* and *ARL*. New Phytol..

