# Supplementary figures and images for: Mutation of ACX1, a Jasmonic Acid Biosynthetic Enzyme, Leads to Petal Degeneration in Chinese Cabbage (Brassica campestris ssp. pekinensis)
Source: Int J Mol Sci. 2019 May 10;20(9):2310. doi: 10.3390/ijms20092310 (PMC6539522; doi:10.3390/ijms20092310)

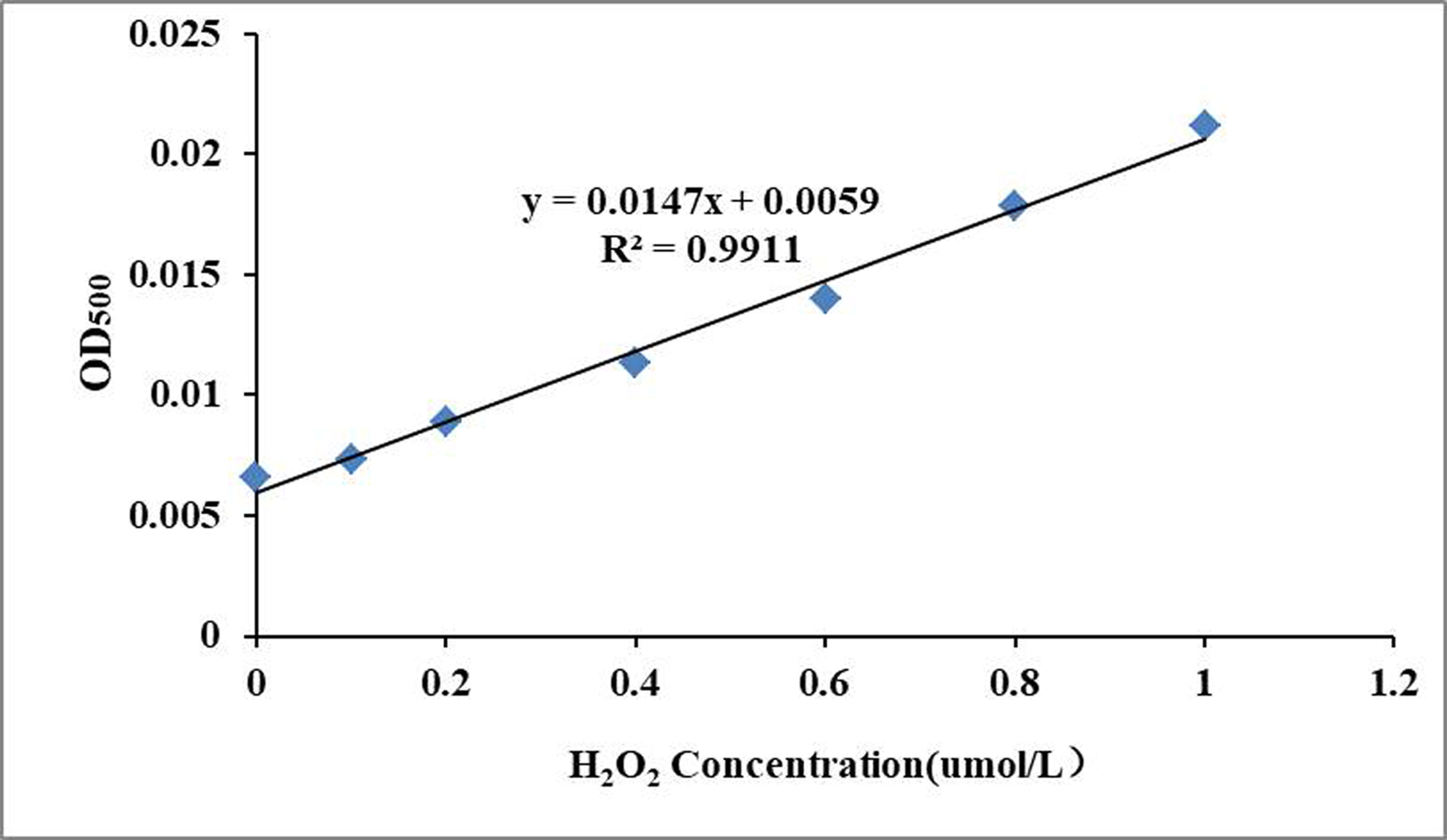

Supplement: Supplementary file 1 [file ijms-20-02310-s001.zip › Supplementary files/Figure S1. A standard curve for H2O2..tif]

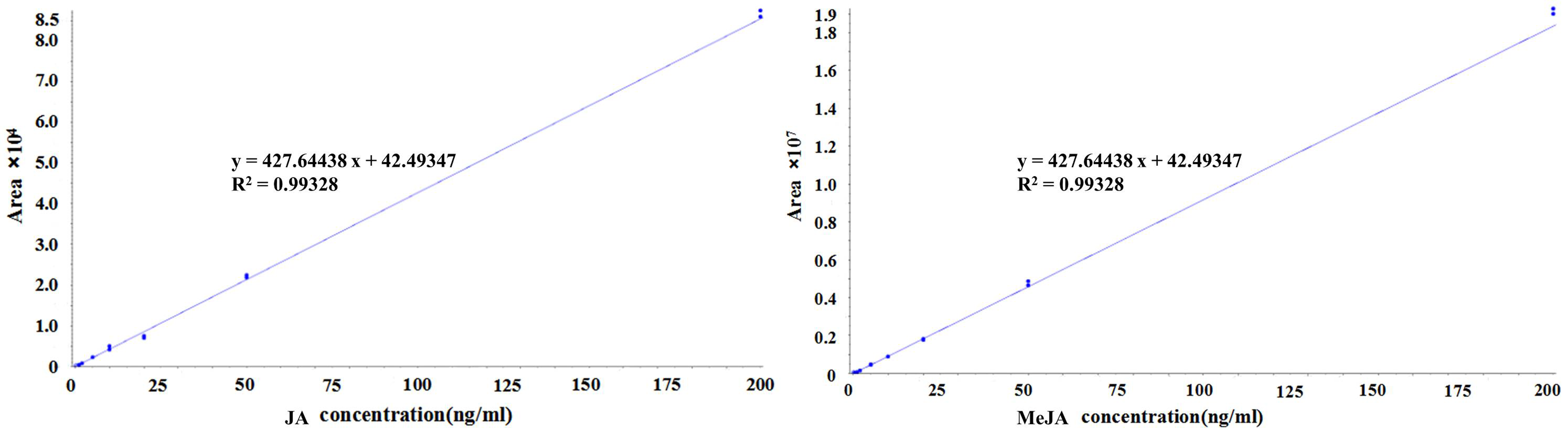

Supplement: Supplementary file 1 [file ijms-20-02310-s001.zip › Supplementary files/Figure S2. Standard curves for jasmonic acid and methyl jasmonate..tif]

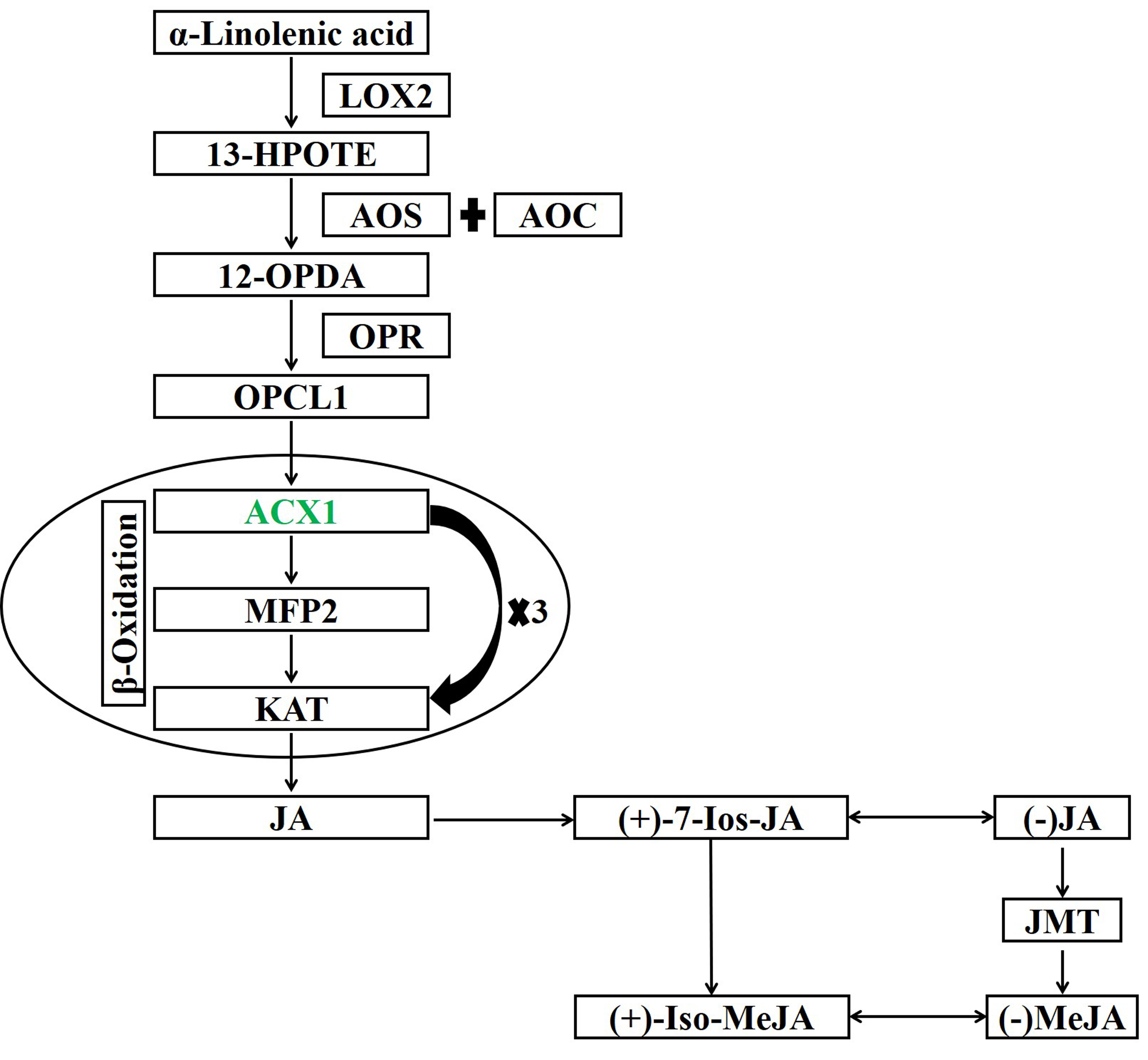

Supplement: Supplementary file 1 [file ijms-20-02310-s001.zip › Supplementary files/Figure S3. Jasmonic acid (JA) biosynthesis in the a┴-linolenic acid metabolic pathway..tif]
